# Synthesis of Chitosan and Ferric-Ion (Fe^3+^)-Doped Brushite Mineral Cancellous Bone Scaffolds

**DOI:** 10.3390/biomimetics9060308

**Published:** 2024-05-21

**Authors:** Lemiha Yildizbakan, Neelam Iqbal, Peter V. Giannoudis, Animesh Jha

**Affiliations:** 1 School of Chemical and Process Engineering, University of Leeds, Leeds LS2 9JT, UK; n.iqbal2@leeds.ac.uk; 2 Academic Department of Trauma and Orthopaedic Surgery, School of Medicine, University of Leeds, Leeds LS2 9JT, UK; p.giannoudis@leeds.ac.uk

**Keywords:** Fe^3+^-doped brushite (dicalcium phosphate dihydrate), chitosan, mechanical properties, scaffold, bone tissue engineering

## Abstract

Biodegradable scaffolds are needed to repair bone defects. To promote the resorption of scaffolds, a large surface area is required to encourage neo-osteogenesis. Herein, we describe the synthesis and freeze-drying methodologies of ferric-ion (Fe^3+^) doped Dicalcium Phosphate Dihydrate mineral (DCPD), also known as brushite, which has been known to favour the in situ condition for osteogenesis. In this investigation, the role of chitosan during the synthesis of DCPD was explored to enhance the antimicrobial, scaffold pore distribution, and mechanical properties post freeze-drying. During the synthesis of DCPD, the calcium nitrate solution was hydrolysed with a predetermined stoichiometric concentration of ammonium phosphate. During the hydrolysis reaction, 10 (mol)% iron (Fe^3+^) nitrate (Fe(NO_3_)_3_) was incorporated, and the DCPD minerals were precipitated (Fe^3+^-DCPD). Chitosan stir-mixed with Fe^3+^-DCPD minerals was freeze-dried to create scaffolds. The structural, microstructural, and mechanical properties of freeze-dried materials were characterized.

## 1. Introduction

Bone is a complex living tissue which experiences metabolic and regenerative disruptions when damaged. Impaired bone healing can be caused by insufficient vasculature, infection, limited cell growth, and scaffold failure, which may lead to non-union [1]. It has been reported that bone tissue regenerates around 1 µm per day, making self-repair nearly impossible for severe defects [2]. In addition, for regrowing a damaged bone, the biomechanical stability for the structural continuity of the tissue is essential for the regeneration process. Bone graft replacement is the most common surgical procedure for filling critical defects (3 cm or more), which is necessary to reduce the need to harvest autografts from patients. Nearly 2 million patients yearly need critical defect void-filling procedures [3]. Allografts and xenografts do not offer a better solution than autografts because of the increased risk of immune response and infection, which may demand revision surgery [4]. However, autograft harvesting has been linked to increased morbidity at the donor site after surgery [5,6]. Engineering a methodology for fabricating scaffolds that supports osteogenesis and angiogenesis might bridge the gap in the growing demand for biocompatible and resorbable scaffolds [7].

Scaffolds are significant in tissue engineering as they promote and guide new tissue development in vivo, performing as a matrix for cell anchoring, inducing particular cellular responses, transporting nutrients and growth factors, and are responsible for cell retention in repairing the defect [8]. An ideal bone scaffold should exhibit biocompatibility, non-toxicity, osteogenic potential to promote new bone growth, load-bearing properties, and a porous structure for nutrient circulation during fracture healing [9]. Natural polymers frequently have highly organised structures that can help cells grow at different phases of development. Crustacean and mushroom-derived chitosan (CS) are widely used biopolymers for various biological purposes, including bone tissue engineering [10]. The chemical structure of chitosan is a linear polysaccharide comprising glucosamine and N-acetyl glucosamine units, connected by β (1–4) glycosidic linkages. CS is a biodegradable polymer with haemostatic characteristics, antibacterial activity and biocompatible properties, which are beneficial for bone tissue engineering applications [11]. CS has shown promise for bone regeneration [12,13], particularly when combined with minerals such as calcium phosphate (CaP) ceramics or apatites [14,15]. It is widely known that CaP improves osteoconductivity, scaffold degradation [16,17] and has been reported to stimulate the adhesion, proliferation, and differentiation of bone marrow mesenchymal stem cells [18,19]. The bioactivity of CaPs has been linked to the composition and structure comparable to the mineral phase of bone [20]. 

Biomaterials utilised in bone implants should potentially resorb, giving way to regenerated bone. At pH 7, the thermodynamic solubility of CaP decreases from dicalcium phosphate dihydrate (DCPD) > octacalcium phosphate (OCP) > tricalcium phosphate (TCP) > hydroxyapatite (HAP), whereby DCPD is the most soluble form amongst the four phosphates [21]. The apparent solubility of DCPD, TCP, and OCP in aqueous media determines the condition for the precipitation of biological apatite precursor for new bone formation and mineralisation [22,23]. The resorption studies on DCPD minerals demonstrate that the mineral exhibits excellent biological characteristics regulating pH locally by making Ca^2+^, HPO_4_^2−^, OH^−^ and H^+^ ions available as starting ingredients in vitro and in vivo environments for new bone formation [24]. By comparison, it was found that when the porous cylindrical HAP scaffolds were implanted into the cancellous bone of rabbits, after six months, it resorbed slowly (~5.4% in volume) [25]. By contrast, the TCP minerals resorbed far more rapidly (85.4% in volume) than HAP under the same pH condition [3], [25]. Because of the chemical bonding, the CaPs are inherently brittle and exhibit poor mechanical properties. However, combining CaPs with ions in the lattice, such as silicon (Si^4+^), zinc (Zn^2+^), iron (Fe^2+^/Fe^3+^), and magnesium (Mg^2+^) [26,27,28] is known to reduce brittleness. Doping DCPD with 10 (mol)% of Fe^2+^ and Fe^3+^ ions yields an optimal combination of biomechanical properties for osteogenesis [26].

This article focuses on Fe^3+^-ion doping DCPD (0 to 50 (wt)%) and fabricating highly porous cancellous freeze-dried CS scaffolds. The scaffolds have been physicochemically and biologically characterised.

## 2. Materials and Methods

### 2.1. Synthetic Cancellous Bone Scaffolds

A 3 (wt)% chitosan stock solution was prepared to fabricate the synthetic cancellous structure needed for bone formation. High molecular weight chitosan flakes (Sigma-Aldrich, CAS: 9012-76-4, Taufkirchen, Germany, 3,100,000–3,750,000 Da, >75% deacetylated) were dissolved in 2 (*v*/*v*)% acetic acid (AcrosOrganics, Geel, Belgium, MFCD00036152) solution under continuous mixing for 24 h.

Fe^3+^-DCPD mineral (Ca_0.9_Fe_0.1_HPO_4_∙2H_2_O) was synthesised via a slow dripping method. In brief, 200 mL of a 0.1 M calcium nitrate (Ca(NO_3_)_2_•4H_2_O) (Fisher Chemicals, CAS: 13477-34-4, Hampton, VA, USA) aqueous solution (A) was heated to 37 °C and 0.83 g of iron (Fe^3+^) nitrate (10 (mol)%) was dissolved into solution (A). Then 200 mL of a 0.1 M (NH_4_)_2_ HPO_4_ (Acros Organics, CAS: 7783-28-0, Geel, Belgium) aqueous solution (B) was added dropwise to the solution (A) under continuous magnetic stirring for 2 h at 37 °C. After mixing solutions (A) and (B), the heat plate and stirrer were switched off. The mixture was allowed to settle for 1 h to allow precipitation. The Fe^3+^-DCPD precipitate was filtered using Whatman Grade 44 filter paper (Merck, WHA1444110, Darmstadt, Germany) and washed three times using distilled water. The collected mineral was placed into a furnace and dried for 24 h at 80 °C.

**Figure 1:**
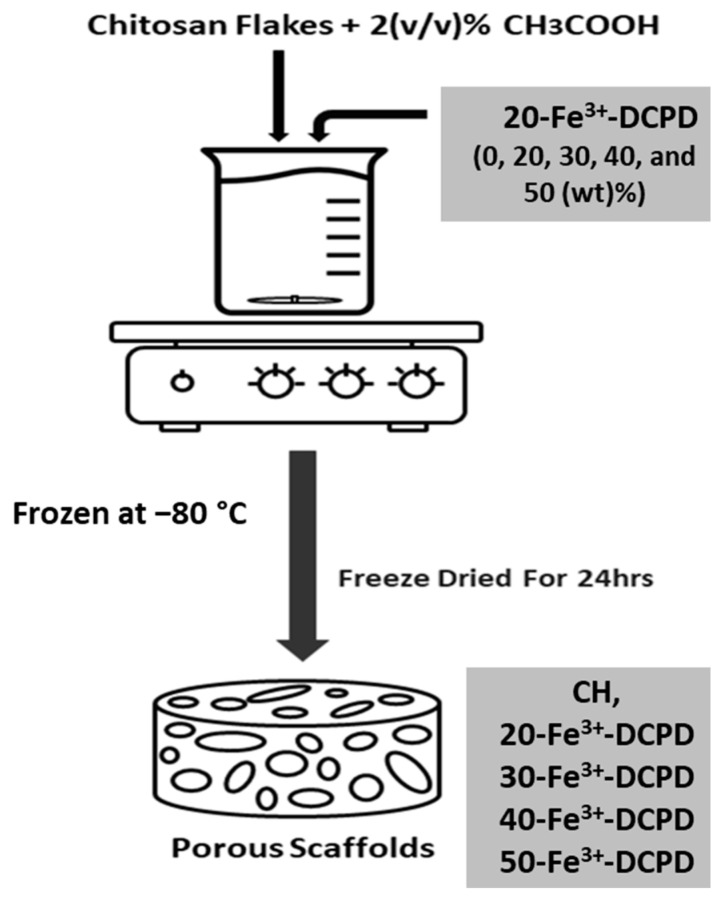
Overview illustration of the freeze-dried scaffolds synthesis process.

The fabrication process of the freeze-dried CS scaffolds is illustrated in Figure 1. The mineral-free and varying concentrations of Fe^3+^-DCPD mineral (20, 30, 40 and 50 (wt)%) loaded CS stock solutions were continuously mixed via a magnetic stirrer for 6 h, producing homogenous suspensions. Measured amounts of unloaded and Fe^3+^-DCPD mineral-loaded CS solutions were frozen at –80 °C for 24 h and then transferred into a freeze drier (VirTis 4 KB ZL Benchtop K (SP Industries, Warminster, PA, USA)) machine at −100 °C and pressure of 43 mTorr for 24 h.

The freeze-dried mineral-free and Fe^3+^-DCPD loaded CS scaffolds were treated with 1 M sodium hydroxide NaOH (Sigma-Aldrich, CAS: 1310-73-2) for 10 min to decrease the CS dissolution rate. After the stipulated duration, the scaffolds were washed five times with deionized water to remove the traces of NaOH. The code names of fabricated freeze-dried scaffolds are displayed in Table 1.

### 2.2. Freeze-Dried Materials Characterisation Techniques

*Raman Spectroscopy:* A Renishaw inVia Raman microscope with a laser excitation source at a 785 nm wavelength and 24.9 mW launch power was used, which, after focusing via ×50 objective lens, was reduced to < 5 mW. The incident power of less than 5 mW was necessary to prevent photo-damage of the samples during spectroscopic analysis. All freeze-dried scaffolds listed in Table 1 were analyzed to ascertain the change in the molecular bonds with the composition of minerals suspended in the CS mixture. The frequency of the vibrational range was from 10 cm^−1^ to 3000 cm^−1^.

*Fourier Transform Infrared Spectroscopy (FTIR):* The Vertex 70 FTIR spectrometer (Billerica, MA, USA), with the attenuated total reflection (ATR) mode, was used to analyse the fabricated freeze-dried scaffolds by molecular vibration spectroscopy analysis. A mid-IR (MIR) lamp was used as the light source, and the beam splitter was KBr. 32 scans were for each scaffold at a spectral resolution of 4 cm^−1^ between 400 cm^−1^ and 4000 cm^−1^.

*X-ray Diffraction (XRD):* Samples were characterized using a D8 X-ray diffractometer with the Cu Kα radiation (λ = 0.15406 nm) in the 5° to 80° Bragg angle (2θ) scanning range with a step size of 0.065 and a scan speed of 0.014° s^−1^. The Rietveld refinement was used to determine the crystallinity of samples using peak shape and intensity analysis, and HighScore Plus software (PANalytica X’Pert HighScore Plus v3.0, Malvern, UK) was used to evaluate recorded patterns.

The average crystallite size distribution in freeze-dried fabricated scaffolds (CH, 20-Fe^3+^-DCPD, 30-Fe^3+^-DCPD, 40-Fe^3+^-DCPD, and 50-Fe^3+^-DCPD and in Fe^3+^-DCPD) were measured using the peak broadening analysis from X-ray diffraction using the Debye-Scherrer equation:

(1) $$D=\frac{K\lambda }{\beta cos(\theta )}$$

In Equation (1), *D* stands for the crystallite size (nm), K is a shape factor constant ~0.9, λ for the wavelength (0.154 nm), β illustrates the half-width of the diffraction band (FWHM) (radians), and (θ) is the Bragg-diffraction angle (peak positions in radians).

*Scanning Electron Microscopy (SEM) and Energy Dispersive X-ray (EDX):* The Hitachi SU8230 1–30 kV cold field emission gun SEM (Düsseldorf, Germany) was used to examine the morphology of the fabricated freeze-dried unloaded and Fe^3+^-DCPD mineral-loaded scaffolds. The samples were coated with 6 µm of iridium before SEM analysis to minimize the charging of the surface to improve image contrast. The SEM micrographs were examined using ImageJ software version 1.41 USA. The SEM images of the samples were opened with ImageJ, and the 60 random scaffold pore sizes were measured for each scaffold. The measured pore diameter was analyzed and graphed using OriginLab 2024, USA.

### 2.3. Bulk Property Analysis of Freeze-Dried Materials

Zeta potential measurements were performed using the Melvern Zetasizer instrument. The Fe-DCPD minerals and chitosan had refractive indices of 1.65 and 1.52, respectively.

The tensile mechanical test was performed for each scaffold composition; the test pieces (n = 3) of freeze-dried scaffolds (CH, 20-Fe^3+^-DCPD, 30-Fe^3+^-DCPD, 40-Fe^3+^-DCPD, and 50-Fe^3+^-DCPD) were tested using the Instron 5569 machine (Norwood, MA, USA). The rectangular samples (5 × 1 cm) were positioned between polystyrene segments to avoid slippage and were tested with a 100 N load cell at a 1 mm/min strain rate without pretension. The tensile strength and Young modulus were assessed using stress-strain graphs and calculated using Equations (2) and (3).

(2) $$Tensile\;Strength=\frac{Force}{Area}=\frac{F}{A}$$

(3) $$Young’s\;Modulus=\frac{Stress}{Strain}=\frac{F*Li}{A*∆L}$$

Here, F denotes the maximum force applied to the scaffold, Li represents the initial length of the scaffold, A shows the cross-sectional area of the fabricated scaffold, which is calculated by width*thickness, and ∆L is the elongation of the scaffold after loading.

Scaffold degradation testing involved drying the samples (n = 3) in a furnace for 24 h at 60 °C and weighed (Wo), then submerged in PBS for 4 weeks at 37 °C. Each scaffold was removed from the PBS solutions (PBS, Life Technologies, Paisley, UK) every week, dried in a furnace at 60 °C for 24 h, and then weighed to determine the weight change. After each recorded measurement, the samples were placed again in fresh PBS solutions. The percentage loss in weight was calculated using Equation (4).

(4) $$Degradation\;\%=\frac{Wo-Wd1}{Wd1}*100$$

Wo is the scaffold’s initial weight, and Wd1 represents the scaffold weight at time (t).

Scaffold swelling testing involved drying (60 °C for 24 h) and weighing (Wd) samples. Next, scaffolds were immersed in the PBS solution for 300 min at 37 °C using Eppendorf tubes. The dried scaffolds were then reweighed on an electronic scale to characterize the swelling characteristics. Equation (5) was used to determine the swelling percentage for each group (n = 3).

(5) $$Swelling\;\%=\frac{Ww-Wd}{Wd}*100$$

$W_{w}$ represents the scaffolds’ wet weight, and $W_{d}$ is the dry weight of the scaffolds.

### 2.4. In Vitro Testing

All scaffolds (CH, 20-Fe^3+^-DCPD, 30-Fe^3+^-DCPD, 40-Fe^3+^-DCPD, and 50-Fe^3+^-DCPD) with diameters of 5 mm and heights of 2.5 mm were sterilized using 70 (*v*/*v*)% ethanol and then washed thrice with PBS.

The Leeds General Infirmary was granted ethical authorization by the Yorkshire and Humberside National Research Ethics Committee to obtain human tissue samples (ethics reference 06/Q1206/127 for bone marrow aspirate (BMA)) from hip replacement patients. The cells were cultured to generate adherent bone marrow mesenchymal stem cells (MSCs). Once confluent, the cells were frozen using 10% DMSO from Thermo Scientific in Loughborough, UK, in 45% Dulbecco’s Modified Eagle Medium (DMEM), Life Technologies, Paisley, UK, and 45% fetal bovine serum (FBS), Thermo Scientific, Loughborough, UK. For in vitro cytotoxicity testing, frozen vials containing cells from three donors were defrosted, pooled, and cultured to passage 3 (p3) in complete MSC StemMACS media (SM) (Miltenyi Biotec, Bisley, UK). Cells were seeded in T25 flasks (Corning, New York, NY, USA) at a density of 2 × 10^5^ and incubated at 37 °C and 5% CO_2_ until almost confluent and ready for use. Half of the media change was performed twice weekly to sustain the cultures. After detaching the cells, the flasks were rinsed with DPBS and treated with 5 mL of Trypsin/ethylene diamine tetra acetic acid (EDTA) from Sigma (Poole, UK) for 5–7 min at 37 °C. Then, 15 mL of DMEM with 10% FBS was added to the flask to cease trypsin activity. A 20 mL cell culture was centrifuged at 1800 rpm for five minutes to produce a pellet. Cells were resuspended in full DMEM media and counted with trypan blue.

***Direct Cytotoxicity tests:*** Sterilized scaffold samples (n = 2) were placed into a 6-well plate and secured with steri-strips (3 M steri-strips, Medisave, UK). Following a seven-day ISO10993-5:2009 (E) part 5 protocol [29], 50,000 cells/well in 2 mL StemMACS were introduced to each well, with a control group comprising just cells without scaffolds with StemMacs. At one, three, and seven days, images of the scaffold/cell interfaces were obtained using an EVOS microscope (ThermoFisher Scientific, Waltham, MA, USA).

***Indirect Cytotoxicity:*** Scaffold eluates were prepared from all types of scaffolds (n = 2 from each scaffold type) according to the ISO standard: ISO 10993-12:2021 [30] part 12. Each sterile scaffold was placed in 6-well plates with 3 mL of SM medium per well to obtain the sample eluates. The plates were then incubated for 72 h, 7, and 14 days at 37 °C and 5% CO_2_. On the day of extract collection, 330 µL of media was collected in 6 Eppendorf tubes from each scaffold. The test settings comprised a positive control (SM), a negative control (10% DMSO in SM), and duplicate extracts from each scaffold. Once the eluate was prepared, cytotoxicity was evaluated according to ISO: 10993-5:2009 (E) part 5: Tests for in vitro cytotoxicity.

The fabricated freeze-dried scaffolds (Fe^3+^-DCPD mineral-free chitosan and different amounts of Fe-DCPD-loaded chitosan scaffolds) were performed in two parts. These are indirect cytotoxicity and cell proliferation by XTT ((2,3-bis-(2-methoxy-4-nitro-5-sulfophenyl)-2H-tetrazolium-5-carboxanilide)) Assay.

***Indirect Cytotoxicity by XTT Assay:*** Three MSCs (n = 3) were pooled and placed in a 96-well plate with 200 µL of SM medium at 1 × 10^4^ cells per well for 24 h. After 24 h, the medium was replaced with 100 µL of defrosted extracts containing the scaffold eluate, negative control, or positive control for another 24 h before adding XTT reagents, as explained below.

***Proliferation assay by XTT assay:*** Three different MSCs were seeded in duplicate (for each scaffold, n = 2 samples were used) into a 96-well plate in 200 μL of SM at 500 cells/well for 24 h. After 24 h incubation, the basal media was removed and replaced with 100 μL/well containing either the scaffold eluate, negative or positive control. Then, the pellet was put in the incubator at 37 °C for 4 days. After four days, the XTT test was performed as indicated above, and the cell proliferation was quantified compared to the positive control.

***XTT Assay:*** XTT cell proliferation assays were performed according to the manufacturer’s instructions. Briefly, 5 mL of XTT labelling reagent (Sigma, Dorset, UK) was mixed with 0.1 mL of electron coupling reagent in a 96-well microplate. Following that, media in wells (scaffold extract, positive or negative control medium) was removed and replaced with 100 µL of DMEM with 10% FBS and 50 µL of XTT solution and incubated at 37 °C for 4 h. The aliquots from each well were transferred to a new plate and read using a microplate reader (Cytation 5, BioTek, Winooski, VT, USA) at 450 and 630 nm (reference wavelengths). The optical density (OD) was estimated by subtracting the reference wavelength (630 nm) from 450 nm. Cell viability or proliferation inhibition was measured by normalizing the ODs of test wells to those of the positive control.

### 2.5. Statistical Analysis

Data from in vitro experiments were analyzed using Graph Pad Prism (version 9.5.0). The data were analyzed using two-way ANOVA with Geisser greenhouse correction. Matching values were stacked across a row in the datasheet. Multiple comparisons were conducted to determine the percentage of viable cells for each scaffold type at each time point.

## 3. Results

### 3.1. Characterisation Results

**Figure 2:**
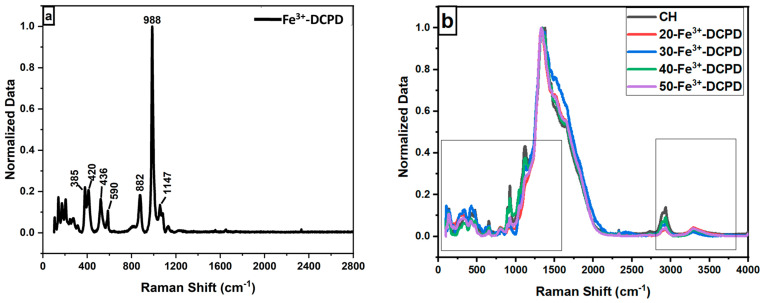

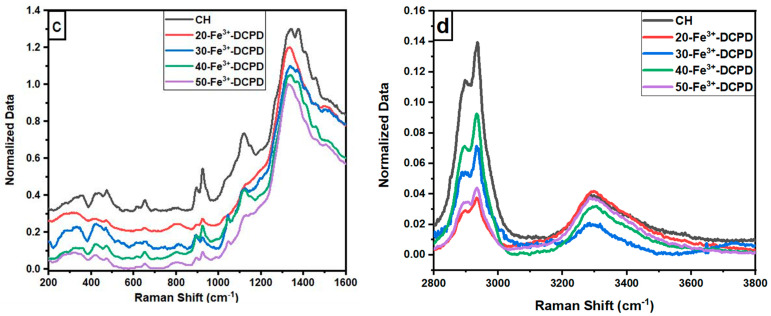
Normalised Raman Spectra for the following: (**a**) Fe-Dicalcium Phosphate Dihydrate mineral (Fe^3+^-DCPD mineral) powder; (**b**) comparison of the various Fe^3+^-DCPD mineral concentrations (0, 20, 30, 40, and 50 (wt)%) loaded chitosan fabricated freeze-dried scaffolds in the range of 100 to 4000 cm^−1^; (**c**) 200 to 2800 cm^−1^ region with 0.2 offsets; and (**d**) 2800 to 3800 cm^−1^.

*Raman Spectroscopy*: The Raman spectroscopic data shows the characteristic vibration bands associated with the functional groups in Fe^3+^-DCPD minerals, mineral-free chitosan (CH) and mineral-loaded chitosan freeze-dried scaffolds (20, 30, 40, and 50-Fe^3+^-DCPD) as illustrated in Figure 1. The Fe^3+^-DCPD mineral exhibits significant phosphate (PO_4_)^3−^ vibrational modes (Figure 2a) [31]. The P-O symmetric stretching of the PO_4_^3−^ ion corresponds to the vibrational mode, which has its highest frequency at 988 cm^−1^. The antisymmetric Raman HPO_4_^2−^ vibration bands are at 882 and 1147 cm^−1^. The lower frequency (PO_4_)^3−^ vibrations are often seen between 385 and 420 cm^−1^, whereas the frequencies between 436 cm^−1^ and 590 cm^−1^ correspond to symmetrical bending of P-O bonds. The vibrational structural analysis demonstrates that adding Fe^2+^/Fe^3+^ ion substitution in DCPD does not change the structure of DCPD, which agrees with similar Raman findings for Fe^3+^-DCPD and DCPD reported in the literature [26,31]. All fabricated freeze-dried scaffolds have exhibited -NH_2_ symmetric and asymmetric stretching from 3200 cm^−1^ to 3450 cm^−1^, corresponding to the CH structure as a part of the N-acetyl glucosamine units. The broad -OH stretching is visible between 3100 cm^−1^ and 3400 cm^−1^, whereas the -CH stretching is present between 2880 cm^−1^ and 2990 cm^−1^. The Raman spectra of mineral-loaded freeze-dried scaffolds are comparable with those of freeze-dried CH scaffolds (Figure 2b–d); however, there is a considerable difference in peak broadenings and intensities in the 2800 cm^−1^ and 3800 cm^−1^ regions. As reported previously [32], the lack of crystallinity of the mineral phase is proportional to the Fe^3+^-DCPD concentration incorporated during the synthesis process, which we have also verified using the X-ray powder diffraction analysis below. For all Fe^3+^-DCPD, the Raman peaks in Figure 2b–d, both below and above 2000 cm^−1^ wave numbers, are significantly broadened which suggests that the presence of Fe^3+^-ions in DCPD structure not only changes the P-O bonding with Ca^2+^ but also introduces more complex (Ca^2+^-PO_4_^3-^-Fe^2+,3+^) interactions, resulting into a more significant distribution of (PO_4_^3−^) vibrational states than without the presence of Fe^3+,2+^ ions. In addition, the vibrational bands for OH^−^ (2800–3000 cm^−1^) and the CH- and -NH_2_ groups are also broadened, as shown in Figure 2c,d. The broader energy distribution of Raman vibrational states confirms that the inorganic phosphate groups are dispersed with the molecular functional groups of chitosan during the synthesis and freeze-drying process. The distribution of vibrational energy of molecular states in the mineralized chitosan confirms that the mineral-chitosan suspension before freeze-drying may also be amenable to changes in the consequential rheology, which we have characterized by determining the zeta potential data below.

### 3.2. Fourier Transform Infrared Spectroscopy

**Figure 3:**
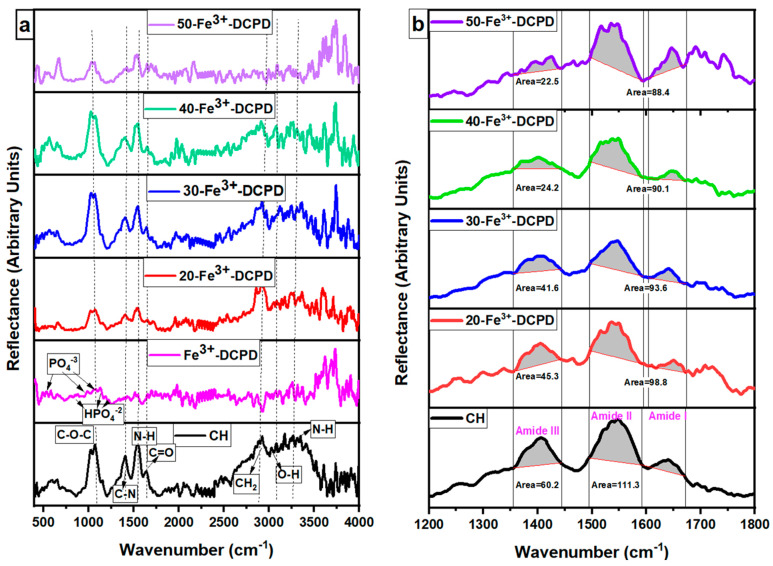
Fabricated freeze-dried chitosan scaffolds were compared with different concentrations of the 10 (mol)% Iron (III) nitrate doped Dicalcium Phosphate Dihydrate (Fe^3+^-DCPD) mineral (CH, 20, 30, 40, and 50-Fe^3+^-DCPD). (**a**) Data were acquired using the Vertex 70 FTIR spectrometer in attenuated total reflection (ATR) mode, between 400 and 4000 cm^−1^, with a resolution of 4 cm^−1^, (**b**) Amide I, II, and III peak comparison.

The FTIR data for fabricated Fe^3+^-DCPD mineral, the unloaded and the Fe^3+^-DCPD mineral-loaded chitosan scaffolds are compared in Figure 3a,b. The FTIR vibration bands 520 cm^−1^, 980 cm^−1^, and 1050 cm^−1^ are associated with PO_4_^3−^ contributions associated with Fe^3+^-DCPD minerals. The bands corresponding to the HPO_4_^−2^ are located at 860 cm^−1^, 1120 cm^−1^, and 1180 cm^−1^, which is consistent with the literature data [32]. The freeze-dried chitosan scaffolds exhibit a broad transmission band at 3291 cm^−1^ and 2993 cm^−1^, corresponding to N-H and O-H stretching, respectively. The peak at 2921 cm^−1^ corresponds to CH_2_ symmetric and asymmetric stretching, and these bands are typical polysaccharide characteristics [33]. The backbone conformation is closely connected to the amide stretching vibration C=O (amide I) at 1645 cm^−1^, whereas the C-N stretching vibration (amide III) is at 1325 cm^−1^. The N-H bending vibration (amide II) occurs at 1550 cm^−1^. The appearance of bands at 1375 cm^−1^ is assigned to the CH_3_ symmetrical deformations, whereas the 1589 cm^−1^ corresponds to the bending vibration of the primary amine N-H bending. The asymmetric stretching of the C-O-C, which is dependent on the crystallinity of chitosan, is responsible for the absorption band at 1153 cm^−1^ [34,35,36,37,38]. The Fe^3+^-DCPD mineral-loaded freeze-dried scaffolds have FTIR spectra identical to the CS scaffold and can be explained by the HPO_4_^2−^ and PO_4_^3−^ peaks associated with the Fe^3+^-DCPD mineral overlapping with the chitosan amide (I, II, and III), CH_3_, and saccharide bands. The Fe^3+^-DCPD mineral phosphate groups (trivalent PO_4_^3−^ and divalent HPO_4_^2−^) and the protonated chitosan amino groups (NH_3_^+^) have been shown to produce strong intermolecular interactions [14]. Additionally, the calculated areas of the amide I, II, and III peaks are shown in Figure 3b, where a noticeable trend reduction in area is observed with increasing concentrations of Fe^3+^-DCPD minerals. As the mineral ratio rises, the divalent (HPO_4_)^2−^ and trivalent (PO_4_)^3−^ groups increase the potential of interaction with protonated CS amino groups, resulting in Coulombic ionic crosslinking reducing the available molecular vibration states and, therefore, the overall peak areas are proportionately reduced [39].

### 3.3. X-ray Diffraction (XRD)

**Figure 4:**
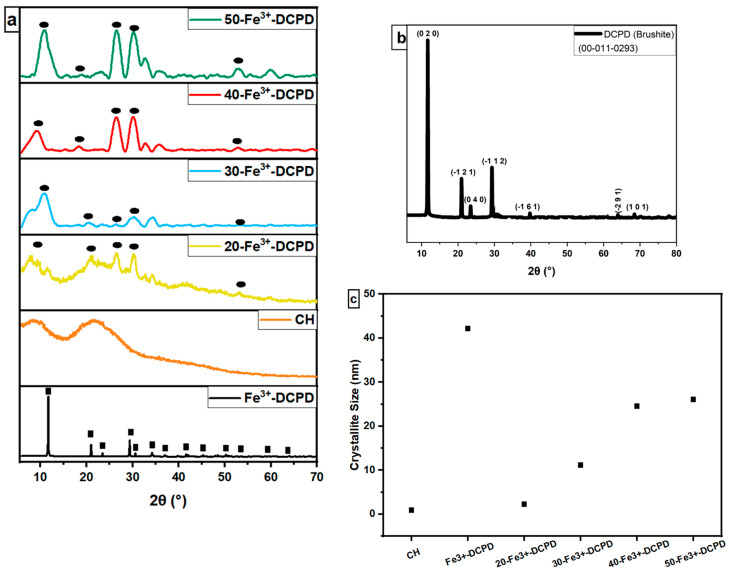
Normalised X-ray diffraction data, (**a**) Experimental XRD spectra for synthesised Fe-DCPD mineral, mineral-free CH, and different amounts of Fe^3+^-DCPD-doped chitosan (20, 30, 40, and 50-Fe^3+^-DCPD) freeze-dried scaffolds, (● corresponds to miller indices (020), (040), (−112), (−231), and (080)). (**b**) DCPD reference spectra (JCPDS), and (**c**) crystallite size comparison. ‘■’ corresponds to the Bragg 2θ diffraction peaks of Fe^3+^-DCPD with miller indices (0 2 0), (0 4 0), (−1 1 2), (−2 3 1), and (0 8 0), respectively.

The experimental XRD diffraction data for the synthesised Fe^3+^-DCPD minerals and freeze-dried scaffolds (CH, 20-Fe^3+^-DCPD, 30-Fe^3+^-DCPD, 40-Fe^3+^-DCPD, and 50-Fe^3+^-DCPD) are compared in Figure 4. Only DCPD peaks were found in a material doped containing 10 (mol) % iron (III) nitrate [26], suggesting no mineral contamination. The Fe^3+^-DCPD pattern’s peaks are comparable with the XRD data for DCPD assembled by the Joint Committee on Powder Diffraction and Standards (JCPDS ref: 00-011-0293). The primary 2θ peaks for the Fe^3+^-DCPD standard are 11.60°, 23.39°, 29.16°, 35.45°, and 47.84°, corresponding to the crystallographic planes (020), (040), (−112), (−231), and (080). Two dominant phases found were DCPD and iron-complexed calcium phosphate mineral, reported elsewhere [26].

The partially crystalline polysaccharide CS exhibits a characteristic XRD fingerprint, comprising two broad peaks at ~10° and ~20°, which relate to the crystal I and II phase forms, respectively [40]. The less hydrated crystal I phase exhibits higher crystallinity. Meanwhile, the more hydrated crystal II phase is amorphous, indicating the intermolecular interactions between the CS polymer chains. The Fe^3+^-DCPD is a highly crystalline mineral due to the Fe^3+,2+^-ion interactions with (HPO_4_)^2−^ and (PO_4_)^3−^ anions in the DCPD lattice. CS phase in Figure 4a appears partially crystalline, which becomes progressively more crystalline as the Fe^3+^-DCPD concentration increases. As a result, a considerable reduction in the CS crystal II phase was observed in all the freeze-dried scaffolds containing the Fe^3+^-DCPD mineral.

Using Scherrer’s Equation (1), the X-ray line broadening data in Figure 4a were analyzed to determine the average crystallite size in the freeze-dried scaffolds. As demonstrated in Figure 4c, the average crystallite size increased as the concentration of Fe^3+^-DCPD mineral increased.

### 3.4. Scanning Electron Microscopy (SEM) and Energy Dispersive X-ray (EDX)

**Figure 5:**
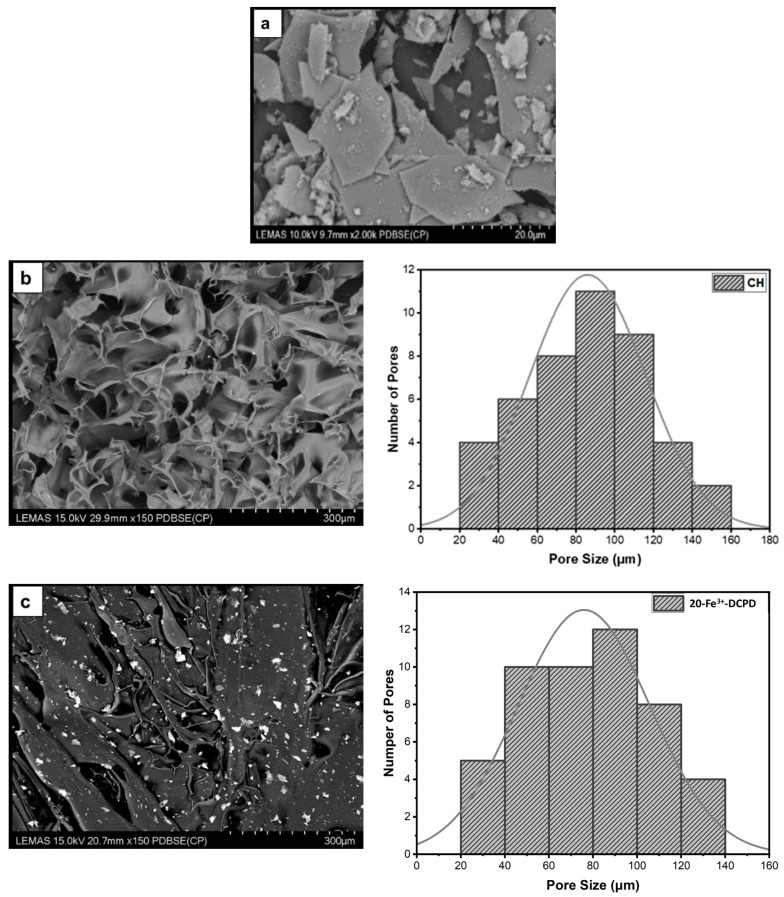

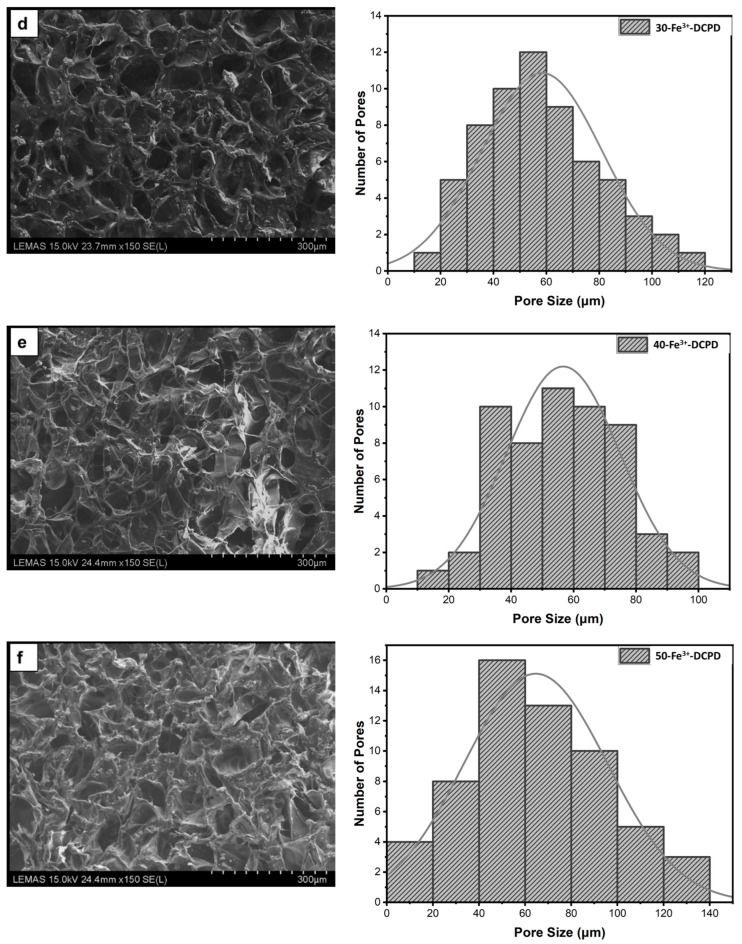
The Hitachi SU8230 SEM image results and corresponding pore size distribution comparisons of (**a**) 10 (mol)% iron (III) nitrate doped Dicalcium Phosphate Dihydrate (Fe^3+^-DCPD) mineral, (**b**) freeze-dried chitosan (CS), (**c**) 20-Fe^3+^-DCPD, (**d**) 30-Fe^3+^-DCPD, (**e**) 40-Fe^3+^-DCPD, and (**f**) 50-Fe^3+^-DCPD.

The microstructures of DCPD minerals, CH, Fe^3+^-DCPD and Fe^3+^-DCDP doped chitosan were investigated via SEM (Figure 5a–d. Increasing Fe^3+^-DCPD concentration led to shape and structural scaffold changes. The mineral-free CH scaffolds show thick lamellae with pores ranging from 20 µm to 180 µm, whereas incorporating Fe^3+^-DCPD minerals reduced the inter lamellae pore size and thickness. The structural alteration and increased number of pores are visible compared to the 20-Fe^3+^-DCPD and 30-Fe^3+^-DCPD scaffolds. In Figure 5e,f, the structure of pores and lamellae are shown for 40-Fe^3+^-DCPD and 50-Fe^3+^-DCPD, respectively, confirming the reduction in the average size of pores and size of lamellae and leading to consequential loss of interconnected pores.

**Figure 6:**
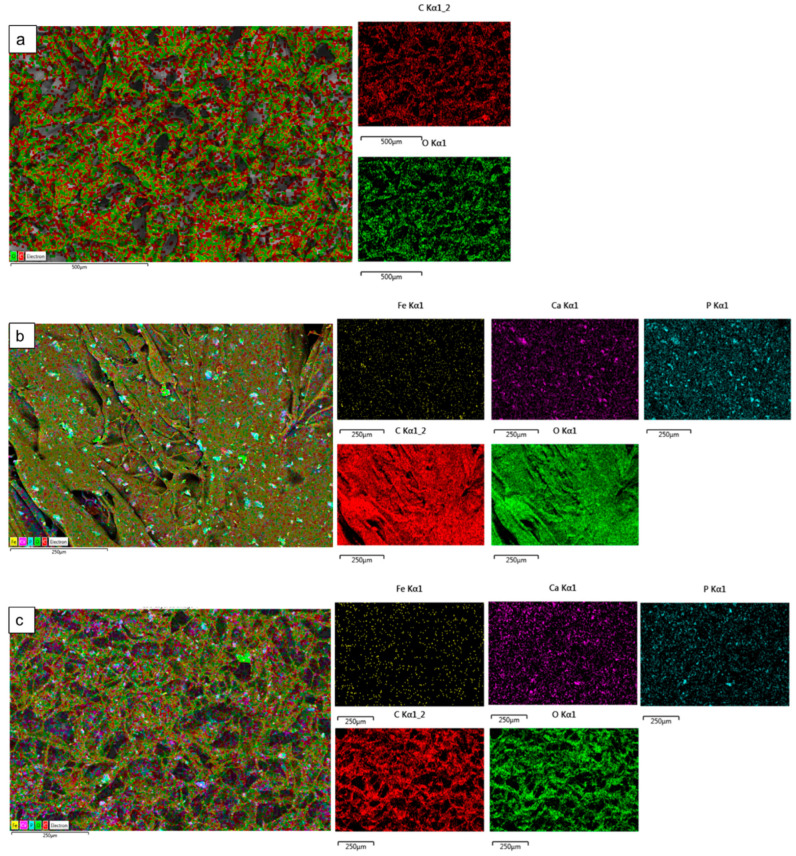

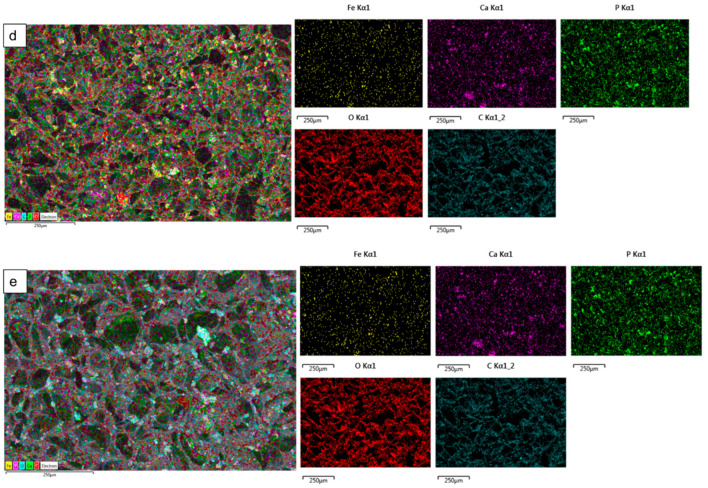
The Hitachi SU8230 SEM with a dispersive energy X-ray (EDX) detector was used to analyse the surface elemental characterization of freeze-dried chitosan scaffolds without Fe^3+^-DCPD mineral and with various Fe^3+^-DCPD mineral concentrations (20, 30, 40, and 50 (wt)%). Freeze-dried (**a**) Chitosan, (**b**) 20-Fe^3+^-DCPD, (**c**) 30-Fe^3+^-DCPD, (**d**) 40-Fe^3+^-DCPD, and (**e**) 50-Fe^3+^-DCPD scaffold. Each colour represents different types of elements in the sample.

The surface elemental analysis results of the fabricated freeze-dried mineral unloaded and loaded chitosan scaffolds (CH, 20-Fe^3+^-DCPD, 30-Fe^3+^-DCPD, 40-Fe^3+^-DCPD, and 50-Fe^3+^-DCPD) are demonstrated in Figure 6a–e. The EDX analysis examined mineral distributions and how increasing amounts of minerals affected pore size. It was found that the distribution of minerals on the surface increased with increased mineral concentration, resulting in varying pore sizes for the samples.

### 3.5. Zeta Potential

The stability of a suspension is characterized by the zeta potential, which measures the potential due to surface charge around the solid surface in colloidal dispersions [41]. The zeta potential of the Fe^3+^-DCPD minerals was found to be −10.53 ± 0.41 mV, implying aggregation, as confirmed by SEM analysis in Figure 5a. The negative surface charge density relates to the presence of phosphate groups (PO_4_^3−^) terminating at the surface of solids.

All the freeze-dried chitosan scaffolds were found to have positive zeta potential values, indicating amino group protonation. Although the magnitude of the potential decreased with the increasing proportion of Fe^3+^-DCPD mineral, the average zeta potential for CH (mineral-free) and 50-Fe^3+^-DCPD mineral-loaded freeze-dried scaffolds decreased from +48.6 ± 0.4 mV to +21.6 ± 0.41 mV, as shown in Table 2. The interaction between the protonated CH scaffold amino groups (-NH_3_^+^) and the Fe^3+^-DCPD phosphate groups (PO_4_^3−^) is likely the reduction in the overall magnitude of positive potential.

### 3.6. Mechanical Test

Increasing Fe^3+^-DCPD concentrations led to a proportionate increase in Young’s modulus and tensile strength compared with mineral-free CH scaffolds (Table 3). The fabricated 30-Fe^3+^-DCPD scaffolds illustrated the most substantial moduli and strength increase compared to the 20-Fe^3+^-DCPD scaffolds. From mineral-free scaffold (CH) to 20-Fe^3+^-DCPD, Young’s modulus increased by 47.5%, while the tensile strength increased by 35%. Young’s modulus increased by 58.3%, and the tensile strength increased by 61.8% when the amount of the Fe^3+^-DCPD mineral rose from 20 to 30 (wt)%. As confirmed by XRD (Figure 4), Fe^3+^-DCPD mineral improved the scaffold’s crystallinity, stabilizing and limiting the CS biopolymer chains.

Scaffolds with smaller average pore diameters exhibited better mechanical characteristics because of the compact structure, reduced pore volume and increased crystalline fractions compared to larger pore sizes. Because scaffold pore sizes varied, the porous scaffolds’ mechanical characteristics were affected. Scaffolds with smaller pore diameters appear to have stronger tensile strengths and can tolerate larger loads than others, possibly because of their denser structure.

### 3.7. Degradation of Scaffolds

**Figure 7:**
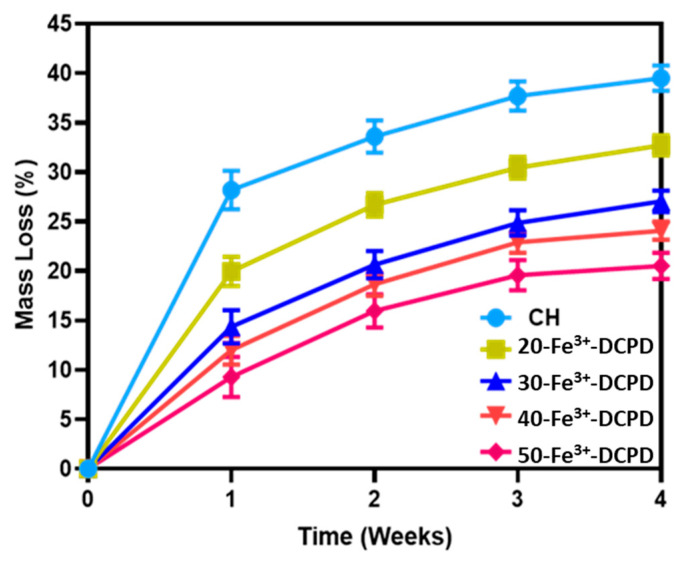
Mineral-free and different concentration Fe^3+^-DCPD mineral embedded chitosan freeze-dried scaffolds degradation results (CH, 20, 30, 40, and 50-Fe^3+^-DCPD) when they are dissolved in phosphate saline buffer (pH 7.4) at 37 °C. Over 4 weeks, the studies were performed in triplicate. The error bars demonstrate each group’s standard deviation (SD) ± mean, n = 3.

The fabricated freeze-dried mineral-free (CH) and different concentrations of Fe^3+^-DCPD mineral (20, 30, 40, and 50 (wt)%) loaded chitosan scaffolds were analyzed for degradation, as shown in Figure 7. The observed patterns show an initial fast degradation rise from 0 to 1st week for all scaffolds. Between the 1st and 3rd weeks, there was a gradual rise, followed by a decrement of degradation rate between 3 and 4 weeks. With increasing Fe^3+^-DCPD concentration, the rate of mass loss was found to decrease with increasing proportion of Fe^3+^-DCPD; the lowest mass loss was found to be 20.5 ± 3.8% for 50-Fe^3+^-DCPD. The degradation rate for CH and other mineral-bearing scaffolds is compared in Figure 6.

### 3.8. Swelling of Scaffolds

**Figure 8:**
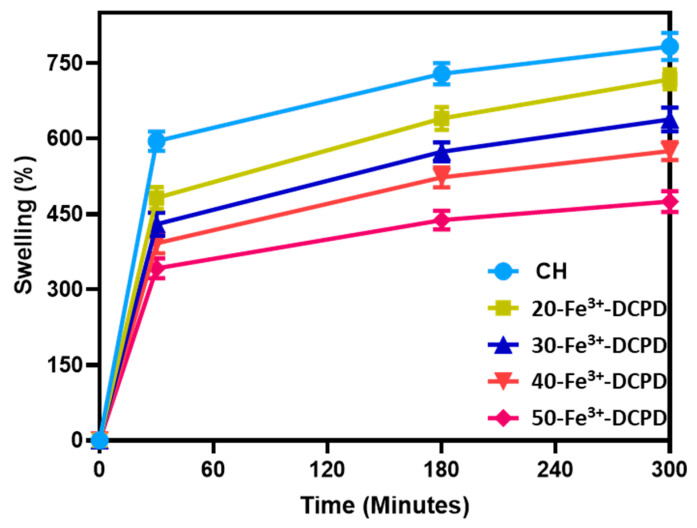
The swelling kinetics of mineral-free chitosan (CH), 20, 30, 40 and 50-Fe^3+^-DCPD freeze-dried scaffolds immersed in phosphate saline buffer (pH 7.4) at physiological temperature 37 °C. Experiments were carried out in triplicates for each of the fabricated scaffolds. The error bars represent the mean ± standard deviation (SD) for n = 3 in each group.

The ability of a scaffold to retain water is a crucial factor in assessing its feasibility for tissue engineering. The swelling properties of scaffolds have been demonstrated to affect cell adhesion, growth, and differentiation considerably [42]. Figure 8 compares the swelling behavior of freeze-dried scaffolds with varying Fe^3+^-DCPD mineral concentrations across time intervals. The observed patterns reveal a rapid increase in swelling from 0 to 30 min for all scaffolds. A slow increase occurs between 30 and 180 min, followed by mass stability between 180 and 300 min. The swelling percentage varies from 783 ± 27.3% for Fe^3+^-DCPD mineral-free CH freeze-dried scaffolds to 475.4 ± 21.8% for 50-Fe^3+^-DCPD mineral freeze-dried scaffolds, demonstrating a considerable influence of Fe^3+^-DCPD mineral concentrations on swelling.

### 3.9. In Vitro Cell Results

#### 3.9.1. Direct Cytotoxicity

**Figure 9:**
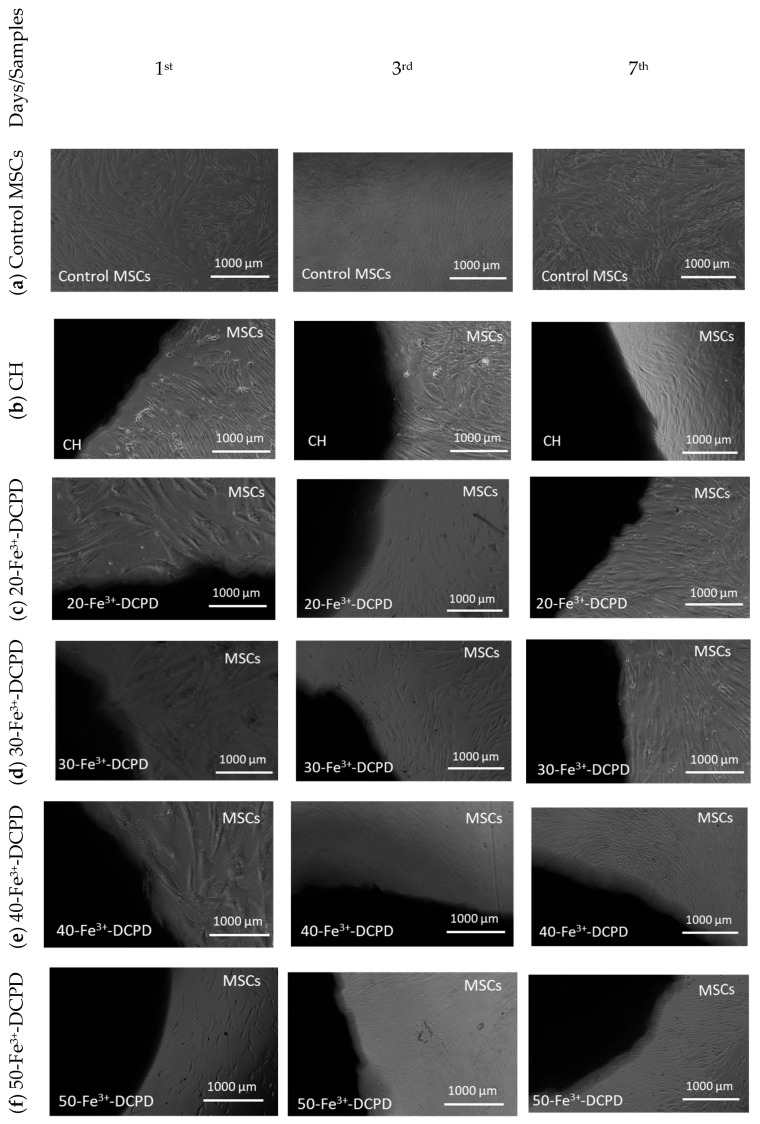
Direct cytotoxicity. Images of chitosan freeze-dried scaffolds with varied Fe^3+^-DCPD concentrations (0, 20, 30, 40, and 50 (wt)%) were obtained at ×4 on the first, third, and seventh days. (**a**) Control MSCs were bone marrow mesenchymal stem cells (MSCs) without scaffolds, (**b**) mineral-free CH scaffold, (**c**) 20-Fe^3+^-DCPD, (**d**) 30-Fe^3+^-DCPD, (**e**) 40-Fe^3+^-DCPD, and (**f**) 50-Fe^3+^-DCPD.

Direct cytotoxicity assay testing is a qualitative evaluation of cytotoxicity that uses microscopic observations to examine the MSC cell shape and environment of media, exhibiting changes in the media’s colour (ISO10993-5:2009). Figure 9 demonstrates the 7-day direct cytotoxicity results. On days 1, 3, and 7, images of the control well with MSCs and the fabricated freeze-dried scaffolds (CH, 20-Fe^3+^-DCPD, 30-Fe^3+^-DCPD, 40-Fe^3+^-DCPD, and 50-Fe^3+^-DCPD) were taken. After day 7, the media’s colour remained comparable to the control; no turbidity indicated a lack of contamination. Moreover, the morphology of the cells at the cell-scaffold interface did not change and remained consistent with the control, indicating that the scaffolds were not cytotoxic to MSCs.

#### 3.9.2. Indirect Cytotoxicity

**Figure 10:**
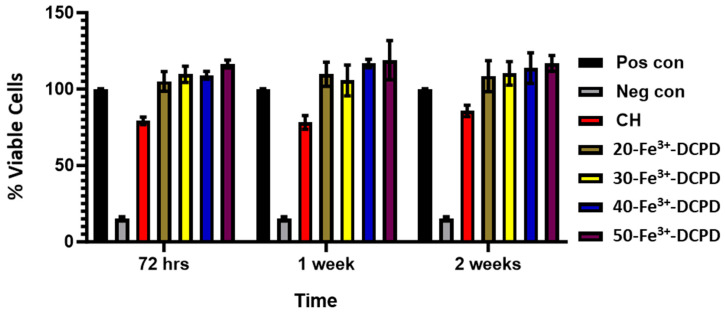
Cytotoxicity by XTT assay test on MSCs exposed to extracts taken from freeze-dried chitosan scaffolds and containing varying concentrations of Fe^3+^-DCPD minerals (20 (wt)% (20-Fe^3+^-DCPD), 30 (wt)% (30-Fe^3+^-DCPD), 40 (wt)% (40-Fe^3+^-DCPD), and 50 (wt)% (50-Fe^3+^-DCPD). The error bars represent the mean ± SD (n = 2 in each group).

**Indirect Cytotoxicity by XTT Assay**: The indirect cytotoxicity findings of the freeze-dried CH and 20, 30, 40, and 50 (wt)% Fe^3+^-DCPD doped chitosan scaffolds (Figure 10) showed greater than 80% cell viability at all periods (3, 7, and 14 days). The indirect cytotoxicity results show that the extract obtained from the scaffolds was non-toxic and presented increased cell viability compared to the positive controls. The results demonstrate Fe^3+^-DCPD minerals did not inhibit cell growth or proliferation. No significant differences were observed between the percentage of living cells exposed to scaffold extract and the positive control MSCs. Furthermore, the findings reveal that all Fe^3+^-DCPD scaffolds promoted MSCs.

**Figure 11:**
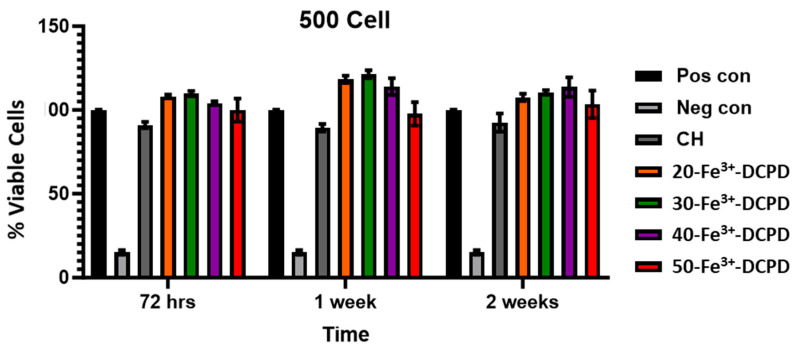
Cell proliferation was assessed using the XTT assay on MSCs exposed to freeze-dried scaffold extracts (CH, 20-Fe^3+^-DCPD, 30-Fe^3+^-DCPD, 40-Fe^3+^-DCPD, and 50-Fe^3+^-DCPD). The fabricated scaffold extracts were seeded 500 cells/well. The error bars represent the mean ± SD (n = 2 in each group). There was no significant difference between freeze-dried scaffolds at all time points.

**Cell Proliferation by XTT Assay**: The MSCs’ proliferative activity was examined by exposing the MSCs to collected scaffold extracts for up to 14 days and then compared with the positive and negative controls (Figure 11). 

Fe^3+^-DCPD minerals enhanced MSC cell proliferation compared to the mineral-free CH scaffold results for each time point. The porous structure of freeze-dried scaffolds allows for the release of significant minerals, which stimulate cellular growth. All Fe-DCPD mineral-loaded chitosan freeze-dried scaffolds (20-Fe^3+^-DCPD, 30-Fe^3+^-DCPD, 40-Fe^3+^-DCPD, and 50-Fe^3+^-DCPD) exhibited increased cell proliferation compared to positive controls at all cell densities. However, compared to the 20, 30, and 40-Fe^3+^-DCPD scaffolds, the 50-Fe^3+^-DCPD scaffolds presented slightly lower MSC proliferation rates. 

## 4. Discussion

The fabricated freeze-dried scaffold chemical structure was revealed using Raman and FTIR spectroscopies. Raman peaks of the Fe^3+^-DCPD [31] and chitosan [43] have been observed similarly to the literature, and the FTIR observations for the samples agree with previously reported data [35,37,38,44].

The human bone cancellous part has a complex, porous structure with non-homogeneous anisotropic properties and porosity ranging from 50% to 90% [42]. The porosity and interconnectivity of the bone scaffold are critical for cell growth and migration, nutrition and waste delivery, and blood vessel invasion [45,46]. Tissue engineering studies have shown that the average pore size of bone scaffolds ranges from 50 µm to 1500 µm [47], [48]. Optimal bone and tissue regeneration requires a minimum pore size range of 50 µm to 100 µm [49,50,51]. Pore sizes of 0 to 50 µm create cellular capsules surrounding the scaffold, limiting cellular waste evacuation and nutrition and leading to necrotic regions in the structure. Large pores (>1500 µm), on the other hand, reduce the scaffold surface area, which limits cellular adhesion [45].

The chitosan suspensions were frozen at −80 °C before freeze-drying as the scaffolds’ initial freezing temperatures influences pore size distribution. Scaffolds frozen at >−80 °C exhibited a lamellar structure with larger holes, whereas those at temperatures less than −80 °C presented compact forms with smaller pores [52]. Rapid freezing, such as via liquid nitrogen, results in 91% to 95% porosity with pore sizes ranging from 13 to 35 µm, which are inadequate for osteoblast development and proliferation [53]. The freeze-drying approach produces bone scaffolds with highly linked porosity structures, as SEM examination results depict. The freeze-dried CH scaffold presented the broadest pore size range. However, when the concentration of Fe^3+^-DCPD minerals rose, the number of pores increased considerably (Figure 5b–f). Pore size distributions reduced, resulting in pore diameters ranging from 20 µm to 140 µm (20-Fe^3+^-DCPD), 10 µm to 120 µm (30-Fe^3+^-DCPD), 10 µm to 100 µm (40-Fe^3+^-DCPD), and 0 µm to 140 µm (50-Fe^3+^-DCPD). Smaller pore diameters (less than 50 µm) have been observed that inhibit cell mobility and capsule formation, leading to necrotic regions owing to inadequate nutrition and waste transport [54,55]. Large pores (>1500 µm) limit cell adhesion and reduce scaffold surface area. Scaffolds with high pore diameters also exhibited restricted mechanical properties due to the increased void volume [56,57,58]. The number of pores associated with the 50-Fe^3+^-DCPD scaffold decreased considerably (Figure 5f) compared to the mineral-loaded scaffolds. The 50-Fe^3+^-DCPD scaffolds on days 3, 7, and 14, MSC cell growth was reduced compared to the CH, 20, 30, and 40-Fe^3+^-DCPD freeze-dried scaffolds likely due to the pores coalescing, resulting in less defined microstructures with lower pore interconnectivity, (Figure 5). The reduced surface area produced by the loss of individual pores and pore interconnectivity, likely resulted in a reduction in the flow of critical nutrients, leading to a reduction in cell proliferation.

The swelling properties of scaffolds have been shown to affect cell adhesion, growth, and differentiation considerably [42]. Synthetic scaffolds with increased water absorption capacities promote cell adhesion; however, their mechanical properties are often reduced [59]. Chitosan is a natural hydrophilic biopolymer [60] that promotes water molecule diffusion due to its structural free volume and the ease with which polymer chains move [61]. As a result, the Fe^3+^-DCPD mineral-free CH scaffolds had the highest liquid absorption, whereas 50-Fe^3+^-DCPD scaffolds had the lowest swelling percentage rise. Swelling percentage experiments indicate that all synthesised scaffolds’ polymer matrixes can swell and retain water, which is favourable for living tissues [62]. The reduction in hydrophilic functional groups in the cationic CH structure, such as the amine (NH_2_) and amide (-CONH, -CONH_2_) groups, is thought to be responsible for the lower equilibrium swelling % in scaffolds incorporating Fe^3+^-DCPD mineral [63].

The bone implant biomaterials should undergo resorption and degradation to allow new bone tissue to develop and replace the implanted material [64]. However, the degradation rate of implanted materials should ideally match the bone osteogenic rate [65,66]. Because of its excellent biological characteristics, quick reaction, and availability of starting ingredients, DCPD has been utilised as a bone-replacing material [24]. DCPD degrades quicker than HAP due to higher crystallinity and Ca/P ratios, making it suitable for usage in bone tissue [20]. Uchida et al. [67] employed HAP as a bone implant to repair the bone after tumour excision in 60 patients at various bone sites; they observed HAP efficiently merged with host bones [67]. The synthesised freeze-dried scaffolds indicate that increasing the Fe^3+^-DCPD concentration reduced the scaffold’s mass loss. The freeze-dried CH scaffold lost the most mass (39.5 ± 1.3%), whereas the 50-Fe^3+^-DCPD scaffold lost the least (20.5 ± 1.8%) after 4 weeks. The XRD analysis demonstrates that the mass loss differential is likely owing to the 50-Fe^3+^-DCPD’s greater crystallinity than the other scaffolds. Crystallinity increases hydrogen bonding and intermolecular interactions between CS biopolymer chains, creating a more compact scaffold structure and lowering water molecules’ accessibility to hydrophilic groups.

It is essential for potential bone scaffolds and the degradation products to be biocompatible, particularly after in vivo implantation, to minimise cytotoxicity and reduce an inflammatory reaction [68]. The freeze-dried scaffolds’ cytotoxicity was assessed using direct and indirect cytotoxicity via XTT assay. The manufactured scaffolds demonstrated no harmful effects. Furthermore, indirect cytotoxicity data from the XTT experiment showed that increasing Fe^3+^-DCPD concentration improves the percentage of cells alive in the scaffold extract.

To facilitate bone tissue regeneration or repair and the restoration of normal biomechanical performance, it is also necessary for the scaffolds used in bone tissue engineering to offer temporary mechanical strength at the site of the defect [69]. Chitosan is an excellent natural polymer to use in bone tissue engineering scaffolds. It is antimicrobial, biocompatible, and biodegradable, aiding wound healing. However, chitosan lacks adequate mechanical strength (poor tensile strength, low fracture stiffness, and compressive strength) required for load-bearing applications [70]. The combination of CS and CaP improved scaffold mechanical qualities; however, CaP exhibits high brittleness, low tensile stress, and low impact resistance [71]. Therefore, to improve the mechanical characteristics of CaP minerals, iron (Fe^2+^/Fe^3+^) was incorporated. Fe^3+^-ions increased the presence of a protein required for cell adhesion in DCPD samples compared to undoped samples [72]. Fe -ions in DCPD could encourage cell growth [73]. Iron is an element that occurs naturally in the human body, and Fe^3+^ is essential for blood haemoglobin, which transports oxygen; therefore, incorporating bone scaffolds provides no danger of toxicity or rejection [74,75]. The Fe^3+^ doping of CaP enhances toughness and durability, promotes bone formation, accelerates healing, and reduces problems [72]. Thus, adding Fe^3+^-DCPD minerals to CS enhanced the mechanical characteristics and increased osteoconduction. 

Increasing the concentration of the Fe^3+^-DCPD mineral (HPO_4_^−^ and PO_4_^3−^ ions) constricts the CS biopolymer chains, resulting in improved mechanical strength. The overall strength of the scaffolds increased with Fe^3+^-DCPD mineral concentrations, where the 50-Fe^3+^-DCPD scaffolds demonstrated a 25.9 ± 0.19 kNm^−2^ increase in Young’s Modulus and a 25.5 ± 0.13 kPa increase in tensile strength. Furthermore, it was found that Fe^3+^-DCPD mineral-doped chitosan scaffolds demonstrated ≥ 20% improved structural strong mechanical characteristics than DCPD mineral-doped chitosan scaffolds [76].

Strong cationic or anionic particles indicate particle stability and dispersion in solution [77,78]. All examined freeze-dried scaffolds exhibited positive (+ve) zeta potentials ranging from +21 to +49 mV. The mineral-free CH scaffold had the highest positive zeta potential (+48.6 ± 0.4), indicating the free protonated amine groups. Adding Fe^3+^-DCPD minerals to chitosan lowered the zeta potential because phosphate ions have an affinity for the protonated amines [77], resulting in the particles’ positive charge. The increase in Fe^3+^-DCPD phosphate ions coincides with reduced zeta potential.

## 5. Conclusions

Incorporating Fe^3+^-DCPD minerals (0 to 50 (wt)%) with CS scaffolds extensive scaffold crystallinity, resulting in stiffer structures with reduced degradation and swelling rates. The mechanical characteristics of 50-Fe^3+^-DCPD scaffolds demonstrated more than five times (31.3 ± 0.06 kN) the mechanical strength compared to mineral-free scaffolds (5.4 ± 0.19 kN). In vitro, results highlight enhanced cell proliferation with increasing Fe^3+^-DCPD mineral concentration at all time points (3, 7, and 14 days) compared to the cellular controls. In summary, the addition of Fe^3+^-DCPD minerals with chitosan improved the scaffolds’ biocompatibility, mechanical characteristics, and percentage (%) cell viability, particularly for scaffolds containing Fe^3+^-DCPD between 30 to 40 (wt)%.

## Figures and Tables

**Table 1:** Summary of the fabricated freeze-dried undoped and Fe^3+^-DCPD doped chitosan scaffolds with code names.

Code	Description	Fe^3+^-DCPD:CH
Fe^3+^-DCPD	10 (mol)% Iron (III) nitrate doped Dicalcium Phosphate Dihydrate	100:0
CH	Chitosan	0:100
20-Fe^3+^-DCPD	20 (wt)% Fe-DCPD mineral-loaded chitosan scaffold	20:80
30-Fe^3+^-DCPD	30 (wt)% Fe-DCPD mineral-loaded chitosan scaffold	30:70
40-Fe^3+^-DCPD	40 (wt)% Fe-DCPD mineral-loaded chitosan scaffold	40:60
50-Fe^3+^-DCPD	50 (wt)% Fe-DCPD mineral-loaded chitosan scaffold	50:50

**Table 2:** Zeta potential values for the mineral Fe^3+^-DCPD, chitosan, and various concentrations of Fe-DCPD-loaded chitosan scaffolds.

Sample	Zeta Potential (mV)	Standard Deviation
CH	+48.6	0.4
Fe^3+^-DCPD	−10.53	0.41
20-Fe^3+^-DCPD	+40.6	0.39
30-Fe^3+^-DCPD	+37.53	0.31
40-Fe^3+^-DCPD	+30.6	0.7
50-Fe^3+^-DCPD	+21.6	0.41

**Table 3:** The Instron 5569 machine mechanical test results of freeze-dried mineral-free and different concentrations of Fe^3+^-DCPD mineral-added chitosan scaffolds (0, 20, 30, 40, and 50 (wt)%, n = 3 samples were used for each type of scaffold).

	CH	20-Fe^3+^-DCPD	30-Fe^3+^-DCPD	40-Fe^3+^-DCPD	50-Fe^3+^-DCPD
Youngs Modulus (kN/m^2^)	5.4 ± 0.19	10.3 ± 0.04	24.7 ± 0.3	30.1 ± 0.51	31.3 ± 0.06
Tensile Strength (kPa)	7.4 ± 0.18	11.4 ± 0.35	29.9 ± 0.28	31.3 ± 0.04	34.9 ± 0.03

## Data Availability

The data supporting this study’s conclusions are available from the corresponding author on reasonable request.
